# Changing environmental conditions have altered the feeding ecology of two keystone Arctic marine predators

**DOI:** 10.1038/s41598-023-39091-9

**Published:** 2023-08-28

**Authors:** Matthew A. Anderson, Aaron T. Fisk, Rodd Laing, Marie Noël, Joey Angnatok, Jane Kirk, Marlene Evans, Liz Pijogge, Tanya M. Brown

**Affiliations:** 1 School of the Environment, University of Windsor, Windsor, ON Canada; 2 Nunatsiavut Government, Nain, NL Canada; 3 Ocean Wise, Vancouver, BC Canada; 4 Putjotik Fisheries, Nain, NL Canada; 5 Environment and Climate Change Canada, Burlington, ON Canada; 6 Environment and Climate Change Canada, Saskatoon, SK Canada; 7 Fisheries and Oceans Canada, West Vancouver, BC Canada

**Keywords:** Ecology, Ecology, Environmental sciences, Ocean sciences

## Abstract

Environmental change in the Arctic has impacted the composition and structure of marine food webs. Tracking feeding ecology changes of culturally-valued Arctic char (*Salvelinus alpinus*) and ringed seals (*Pusa hispida*) can provide an indication of the ecological significance of climate change in a vulnerable region. We characterized how changes in sea ice conditions, sea surface temperature (SST), and primary productivity affected the feeding ecology of these two keystone species over a 13- and 18-year period, respectively, in northern Labrador, Canada. Arctic char fed consistently on pelagic resources (δ^13^C) but shifted over time to feeding at a higher trophic level (δ^15^N) and on more marine/offshore resources (δ^34^S), which correlated with decreases in chlorophyll *a* concentration. A reduction in Arctic char condition factor and lipid content was associated with higher trophic position. Ringed seals also shifted to feeding at a higher trophic level, but on more pelagic resources, which was associated with lower SST and higher chlorophyll *a* concentrations. Years with abnormally high SSTs and reduced sea ice concentrations resulted in large isotopic niche sizes for both species, suggesting abrupt change can result in more variable feeding. Changes in abundance and distribution of species long valued by the Inuit of Labrador could diminish food security.

## Introduction

Climate change indicators over the last few decades reveal that Arctic ecosystems are changing at an increasing rate and into an unprecedented state^1–6^. Warming in the Arctic is occurring at a rate nearly 4 times faster than the global average^1^ leading to accelerating declines in average annual sea ice concentration, extent, and thickness^2^. Increased air temperatures and reduced sea ice are influencing other environmental factors, including sea surface temperature and primary productivity that are leading to alterations in Arctic marine food webs and animals ranges shifting northward^3–5^. Due to these changes, the Arctic is expected to experience a species turnover five times greater than the global average^6^.

Arctic marine food web dynamics are largely driven by sea ice conditions that impact primary production and biogeochemical processes^7,8^. The timing of sea ice break-up and the subsequent spring phytoplankton blooms initiates a period of high primary productivity that drives energy transfer throughout the food web during the summer months. Mid to upper trophic level species, such as Arctic char (*Salvelinus alpinus*) and ringed seals (*Pusa hispida*), rely heavily on the production supported by these spring phytoplankton blooms for periods of intense summer feeding^9,10^. Changes in sea ice conditions can therefore impact the feeding behavior of species that are intrinsically dependent upon the productivity resulting from sea ice break up, including Hudson Bay ringed seals and polar bears (*Ursus maritimus*)^11^. Climate change has also allowed for the northward expansion of forage fish, such as capelin (*Mallotus villosus*), which, are increasingly reported in the diets of both Arctic char and ringed seal^12,13^ as well as arctic seabirds, such as thick-billed murres (*Uria lomvia*)^14^. The geographic range expansion of capelin has reduced beluga whale (*Delphinapterus leucas*) consumption of Greenland halibut (*Reinhardtius hippoglossoides*) in favor of capelin during the summer months^15^, highlighting the complex nature of food web impacts associated with the intrusion of new species.

Arctic char and ringed seals are good indicators of ecological change in the Arctic because of their circumpolar distribution, high abundance, and their sensitivity to environmental conditions^16,17^. These species are also key components for the dietary, economic, and cultural needs of Inuit throughout the Arctic^18,19^. Sea ice conditions are important to both species as ringed seals utilize spring sea ice for molting, reproduction, and raising of their pups, while anadromous, iteroparous Arctic char migrate to the marine environment following spring sea ice break up to take advantage of favorable summer feeding, returning to the same freshwater systems in late summer/early fall to spawn and overwinter^20,21^. Sea ice break up initiates a period of intense feeding that is critical for the condition and survival of Arctic char and ringed seals, as they integrate lipids to prepare for winter and reproduction^9,10^. However, mismatches in timing have resulted in altered zooplankton production in response to earlier sea ice melt and phytoplankton blooms, which affects the foraging behavior of Arctic char and ringed seals, as well as their prey^22–27^.

Arctic char are a diverse species that consist of populations with varying life histories and variable feeding behavior^28^. In the marine environment they are opportunistic feeders with adaptable diets based on available prey, such as pelagic invertebrates and fish^29^. Much like Arctic char, ringed seals are known to have a diverse diet consisting of fish, pelagic invertebrates, and bivalves^30,31^. Ringed seal diets vary throughout the Arctic based on studies using dietary tracers^32,33^ and shifts in their feeding ecology to more pelagic energy pathways have been linked to changing environmental conditions^10,33^.

Stable isotopes of carbon (δ^13^C) and nitrogen (δ^15^N) are commonly used to make inferences about a consumer’s diet in aquatic systems. Based on relative isotopic fractionation processes, δ^15^N can be used to quantify trophic position of an organism, as it shows an increase of approximately + 3.8‰ per trophic level in Arctic systems^34^. Whereas δ^13^C can be used to infer food web carbon sources and relative contributions of inshore/benthic versus offshore/pelagic feeding preferences^35^. Similar to δ^13^C, sulfur stable isotopes (δ^34^S) do not have a large increase between prey and consumer but do vary between habitat and carbon source and are particularly useful in distinguishing between marine versus freshwater resources^36,37^. The use of two or more stable isotopes allows for the opportunity to look at isotopic niche size, which, can provide insight into the variability in the feeding behavior of a species or population over space or time^38^.

Isotopic incorporation rates (i.e., turnover) vary among tissues according to their metabolic activity. For example, tissues with high incorporation rates such as liver, blood, plasma, and fat track isotopic changes in diet closely (days to weeks), whereas tissues with low incorporation rates such as muscle integrate the isotopic signature over a longer time period, tracking the diet over a period of weeks to months^39^. On the other hand, inert tissues (e.g., feather and claws), incorporate the dietary signal when they are synthesized^40^. While the majority of studies on fish and marine mammals primarily analyze isotopic ratios in muscle tissue, seal claws provide a unique opportunity to temporally quantify diet by analyzing growth annuli along the claw, where alternating light and dark annuli correspond to seasonal growth or a 4-to-8-month period^41^, respectively. Ringed seal claws can include up to ten years of growth bands before tips begin being worn down. Once stable isotopes have been assimilated into claw bands, they remain unchanged^42^.

Given the increasing pace of environmental change in the Arctic, there is a need to better understand how this will impact important indicator species^43,44^, as well as marine food web structure and function^45^. There are a broad range of species undergoing dietary shifts that reflect the environmental and ecological change occurring in the Arctic. In this study, we assessed how changes in sea ice conditions, sea surface temperature (SST), and primary productivity influenced the diet of Arctic char over a 13-year period (2006–2019) and ringed seals over an 18-year period (2002–2020) in northern Labrador, Canada. First, we characterized the temporal dietary trends in both species using a combination of δ^13^C, δ^15^N, and δ^34^S in Arctic char muscle and ringed seal claws. We then assessed the influence of sea ice break up, SST, and primary production (chlorophyll *a*) on these dietary changes over time. Finally, to determine how Arctic char health was being impacted by environmental change, we assessed whether changes in diet and environmental conditions had impacted their condition and lipid content..

**Figure 1:**
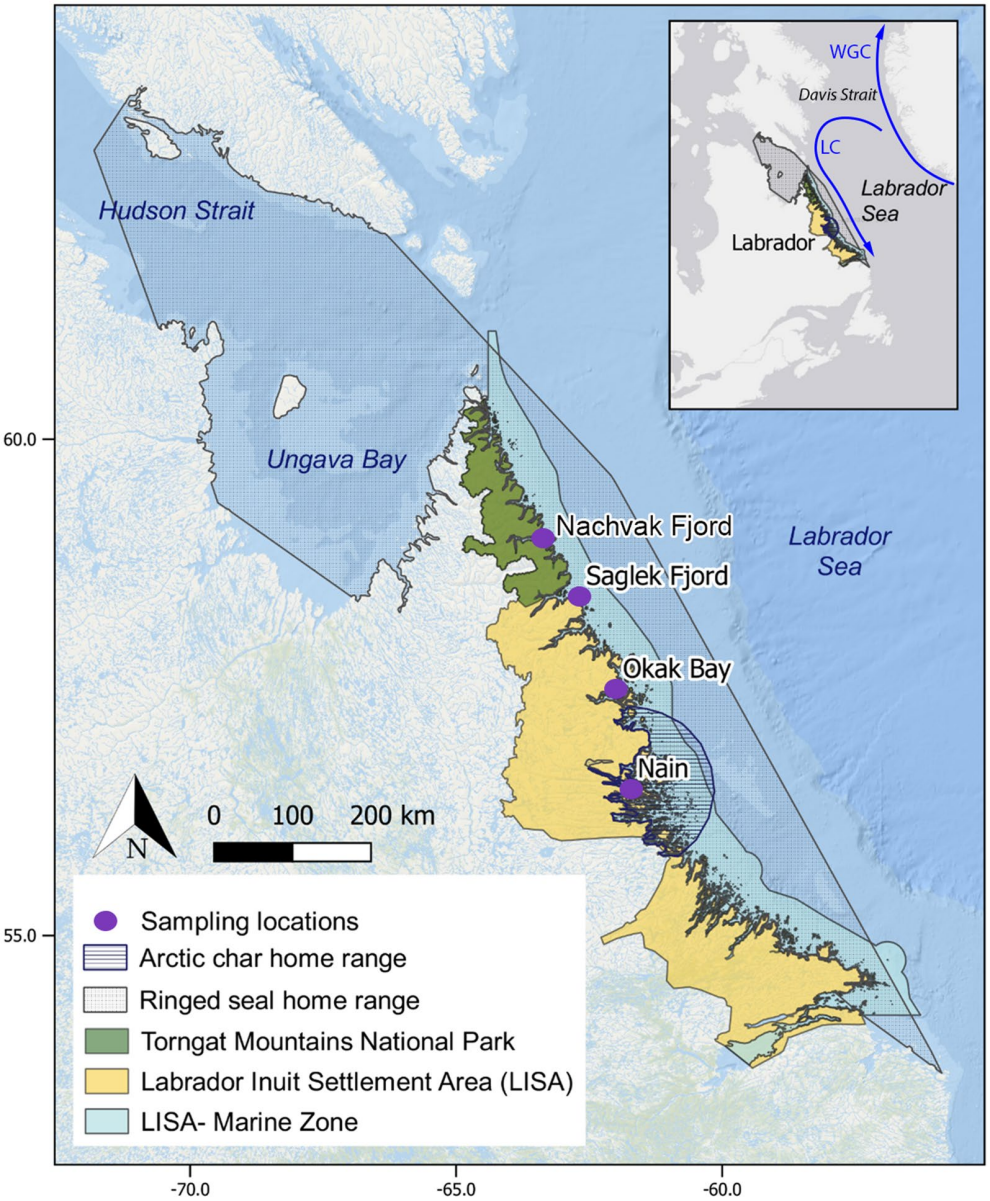
Map of study locations, where Arctic char (Nain) and ringed seals (Nain, Okak Bay, and Saglek fjord) were collected and the location of the Labrador Inuit Settlement Area (LISA), LISA marine zone, and Torngat Mountains National Park. WGC is the West Greenland Current and LC is the Labrador Current. Home ranges for Arctic char and ringed seals are shown and are as described in^65^ and^66^, respectively. The map was produced using QGIS 3.16.3 Hanover.

Our study took place in the Labrador Sea which represents a transition zone between the Arctic and Subarctic and may provide insights regarding what will occur at higher latitudes where Arctic char and ringed seals are also present. This region is influenced by the northwestern Labrador Sea, an area under the influence of both the cold southward flowing Labrador Current and a recirculation cell of the North Atlantic and West Greenland Current^46^. The study area was distributed along the coast of the Labrador Inuit Settlement Area (LISA) which comprises the majority of Labrador’s North Coast (Fig. 1) and is a region that is heavily utilized by the Inuit for hunting and fishing practices.

## Results

### Influence of biological characteristics on Arctic char stable isotopes

There were no differences in length (p = 0.83), weight (p = 0.61), δ^13^C (p = 0.49), or δ^15^N (p = 0.09) between male and female Arctic char. Condition factor and δ^34^S differed between males and females, however these results were influenced by 2014 and 2016 when only female char were collected. When 2014 and 2016 were removed, condition factor (p = 0.09; t-test) did not differ by sex, whereas δ^34^S in females (17.0 ± 0.1 ‰) (mean ± 1 SE) was higher than in males (16.8 ± 0.04 ‰) (t-test, p = 0.04). However, when analyzing individual years, δ^34^S did not differ between males and females. Based on these results, male and female Arctic char were grouped together prior to further analysis. Arctic char fork length and round weight varied by year (ANOVA, both p < 0.0001) with 2006 having the smallest fork lengths (342 ± 21 mm) and round weights (463 ± 91 g), and 2010 having the largest lengths (527 ± 45 mm) and weights (2157 ± 574 g) (Table 1). No relationship was found among the three stable isotopes (δ^13^C, δ^15^N, and δ^34^S) and age or body size for Arctic char (linear regression, p > 0.05).

**Table 1:** Morphometric data, age, stable isotopes, isotopic niche size, percent lipid (mean ± SD, except isotopic niche volume) for Arctic char collected from Nain, Labrador, Canada.

Year	N	Fork length (mm)	Round weight (g)	Condition factor	Age	δ^13^C	δ^15^N	δ^34^S	Isotopic niche volume (‰^3^)	Lipid (%)
2006	19	342 ± 21	463 ± 92	1.1 ± 0.1	4.9 ± 0.9	−19.0 ± 0.2	14.7 ± 0.3	16.6 ± 0.2	1.36	8.0 ± 2.4
2007	20	473 ± 48	1509 ± 512	1.4 ± 0.1	7.7 ± 1.9	−18.6 ± 0.9	14.2 ± 0.4	16.5 ± 0.2	5.58	8.7 ± 2.9
2008	18	491 ± 45	1663 ± 474	1.4 ± 0.2	7.9 ± 2.3	−19.1 ± 0.6	14.1 ± 0.4	16.8 ± 0.2	3.36	9.1 ± 2.1
2009	20	515 ± 61	1746 ± 703	1.2 ± 0.2	7.5 ± 2.3	−21.4 ± 4.3	13.6 ± 1.2	16.6 ± 0.3	74.1	8.7 ± 1.8
2010	18	527 ± 45	2157 ± 574	1.5 ± 0.1	6.9 ± 0.7	−25.5 ± 3.4	13.8 ± 1.3	16.7 ± 0.3	94.3	8.6 ± 4.0
2013	22	363 ± 48	606 ± 242	1.2 ± 0.1	4.8 ± 1.4	−19.9 ± 0.6	14.5 ± 0.4	16.9 ± 0.3	5.80	9.4 ± 2.3
2014	19	454 ± 49	943 ± 200	1.0 ± 0.2	4.7 ± 1.3	−19.9 ± 0.5	14.1 ± 0.4	17.2 ± 0.2	2.46	7.9 ± 1.4
2015	19	446 ± 47	1059 ± 456	1.2 ± 0.2	5.6 ± 1.0	−21.7 ± 0.4	14.8 ± 0.7	17.2 ± 0.3	7.55	9.8 ± 2.7
2016	17	413 ± 62	801 ± 370	1.1 ± 0.1	5.7 ± 1.2	−19.7 ± 0.4	15.1 ± 0.4	17.0 ± 0.3	2.10	9.1 ± 2.9
2017	10	367 ± 81	582 ± 318	1.1 ± 0.2	6.2 ± 1.0	−19.6 ± 0.5	15.1 ± 0.3	16.9 ± 0.3	2.38	5.6 ± 1.0
2018	18	434 ± 62	953 ± 384	1.1 ± 0.1	7.6 ± 1.7	−19.4 ± 0.4	15.1 ± 0.4	17.2 ± 0.5	5.03	7.4 ± 0.7
2019	14	451 ± 77	1057 ± 479	1.1 ± 0.1	6.1 ± 1.2	−19.1 ± 0.3	15.2 ± 0.5	17.3 ± 0.2	1.22	6.7 ± 2.0

### Influence of biological characteristics on ringed seal stable isotopes

No differences were found between ringed seal sample location and δ^13^C (p > 0.05) or δ^15^N (p > 0.05), thus locations were grouped together. Ringed seal sex did not have a significant influence on stable isotopes (δ^13^C or δ^15^N, p > 0.05) thus males and females were grouped together. No relationship was observed between seal age and δ^13^C (p = 0.34), but there was a positive relationship between age and δ^15^N (p = 0.003, R^2^ = 0.03), so all δ^15^N were age adjusted.

### Isotopic niche sizes

**Figure 2:**
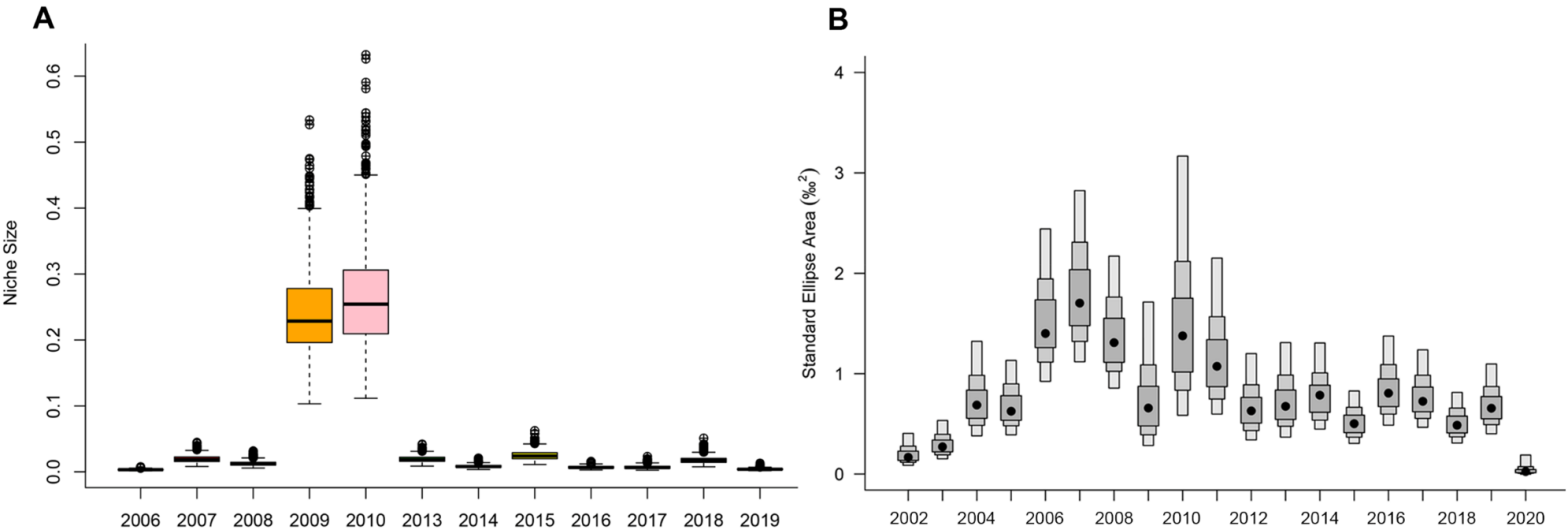
Arctic char isotopic niche volume varied across years with 2009 and 2010 having noteably larger niche volumes compared to other years (**A**). Ringed seal isotopic niche area varied across years (**B**).

Arctic char isotopic niche volume varied by year (ANOVA, p < 0.0001) (Table 1). Of note, Arctic char isotopic niche volume was higher in 2009 and 2010 with respective sizes of 74.1‰^3^ and 94.3‰^3^, compared to all other years (mean = 3.7 ± 2.2‰^3^) (Fig. 2A). Ringed seal isotopic niche area varied by year (ANOVA, p < 0.0001) from a minimum in 2002 of 0.22‰^2^ to a maximum of 1.91‰^2^ ± 0.48 in 2007 (Table 2, Fig. 2B).

**Table 2:** Mean (± SD) stable isotope and isotopic niche area (SEA_c_) for bands of ringed seal claws collected in 2008–2011 and 2019–2020 along the Labrador coast. Stable isotope values for seals were determined by analyzing the claw band that corresponds to that year.

Year	N	δ^13^C	δ^15^N	SEAc ‰^2^
2002	8	−15.9 ± 0.2	13.2 ± 0.4	0.22
2003	11	−15.8 ± 0.2	13.6 ± 0.4	0.33
2004	11	−15.9 ± 0.3	13.6 ± 0.8	0.80
2005	15	−16.0 ± 0.4	13.8 ± 0.6	0.74
2006	18	−16.0 ± 0.6	13.9 ± 0.9	1.63
2007	19	−16.1 ± 0.6	14.0 ± 0.9	1.91
2008	19	−16.3 ± 0.6	14.2 ± 0.8	1.47
2009	6	−16.7 ± 0.5	14.0 ± 0.7	0.70
2010	7	−16.2 ± 0.6	14.0 ± 0.9	1.74
2011	12	−16.2 ± 0.7	14.0 ± 0.9	1.23
2012	11	−15.8 ± 0.3	14.0 ± 0.8	0.69
2013	11	−16.1 ± 0.4	14.3 ± 0.8	0.75
2014	14	−16.3 ± 0.4	14.0 ± 0.8	0.80
2015	16	−16.3 ± 0.3	14.1 ± 0.7	0.52
2016	16	−16.4 ± 0.4	13.9 ± 0.7	0.88
2017	17	−16.4 ± 0.4	14.1 ± 0.8	0.79
2018	17	−16.4 ± 0.3	14.0 ± 0.8	0.52
2019	18	−16.9 ± 0.3	14.2 ± 0.7	0.72
2020	4	−17.0 ± 0.1	14.7 ± 0.4	–

### Temporal variations in diet and condition

**Figure 3:**
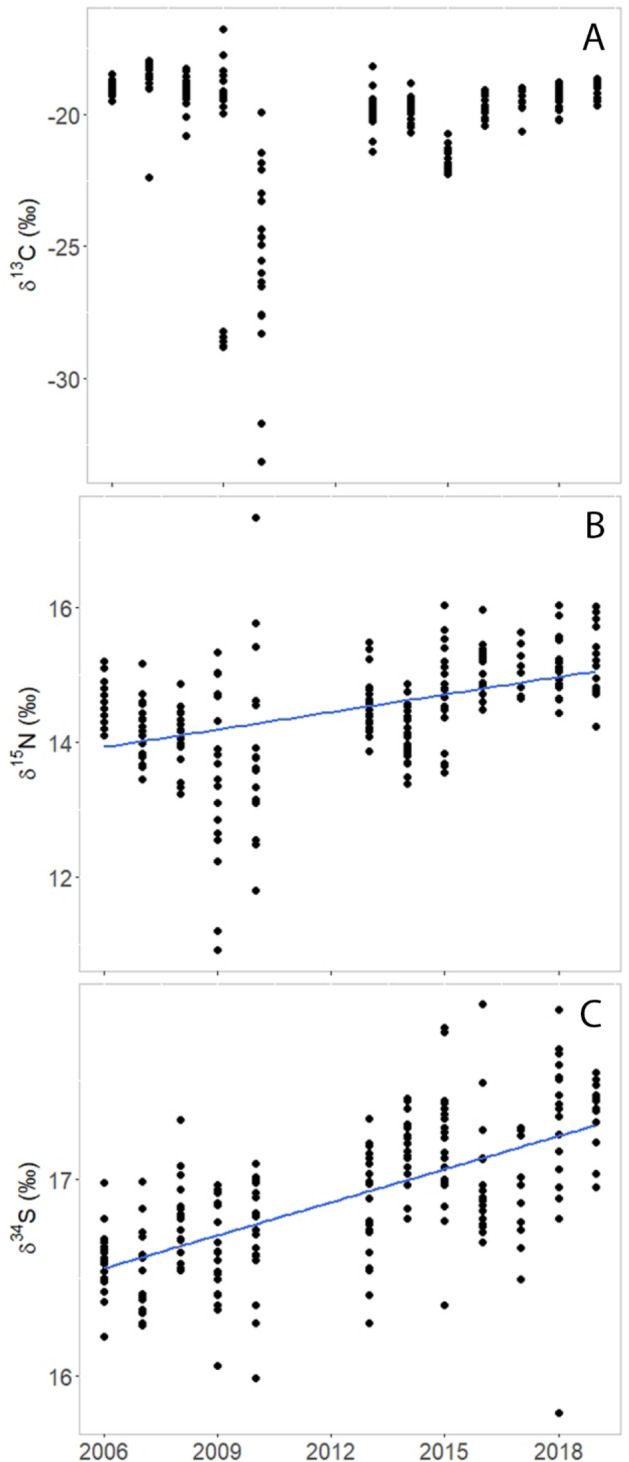
There was no temporal trend in δ^13^C (**A**) but increasing trends in δ^15^N (p < 0.0001; R^2^ = 0.18) (**B**) and δ^34^S (p < 0.0001; R^2^ = 0.41) (**C**) were evident in Arctic char muscle from Nain, Labrador, Canada over time (2006 to 2019).

**Figure 4:**
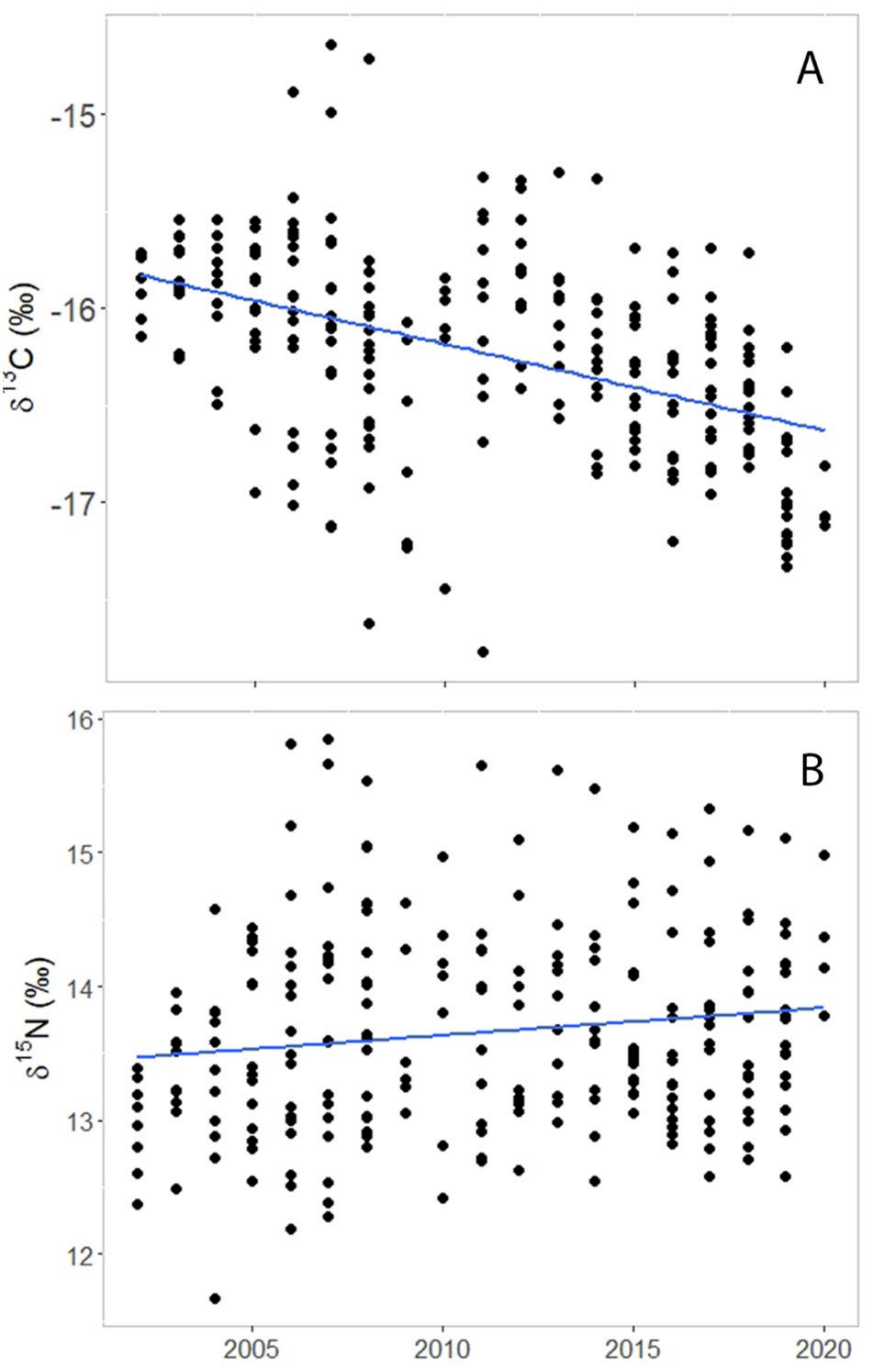
Stable isotope profiles in ringed seals harvested along the Labrador coast revealed that (**A**) δ^13^C decreased (p < 0.0001; R^2^ = 0.21) and (**B**) δ^15^N increased (p = 0.02; R^2^ = 0.02) between 2002 and 2020, indicating that seals have shifted to feeding more pelagically and at a higher trophic position over the study period.

No differences (t-test, p = 0.66) were found in baseline isotopic values for δ^15^N between 2010 and 2020, therefore no corrections were made to δ^15^N. We found no temporal trend in Arctic char δ^13^C (linear regression, p = 0.87) or isotopic niche volume (linear regression, p = 0.30), but δ^15^N and δ^34^S increased with year (p < 0.0001, R^2^ = 0.18; p < 0.0001, R^2^ = 0.41, respectively) (Fig. 3). Arctic char condition factor and percent lipid had a negative relationship with year (p < 0.0001, R^2^ = 0.19 and p = 0.02, R^2^ = 0.02, respectively). Ringed seal δ^13^C values decreased over time (p < 0.0001, R^2^ = 0.21) and δ^15^N had increased over time (p = 0.02, R^2^ = 0.02), whereas isotopic niche area had no temporal trends (p = 0.69) (Fig. 4). Ringed seal condition factor was greater in 2019 (84.5 ± 0.8) than in 2008 (75.1 ± 1.2).

### Influence of environmental change on feeding behavior

**Figure 5:**
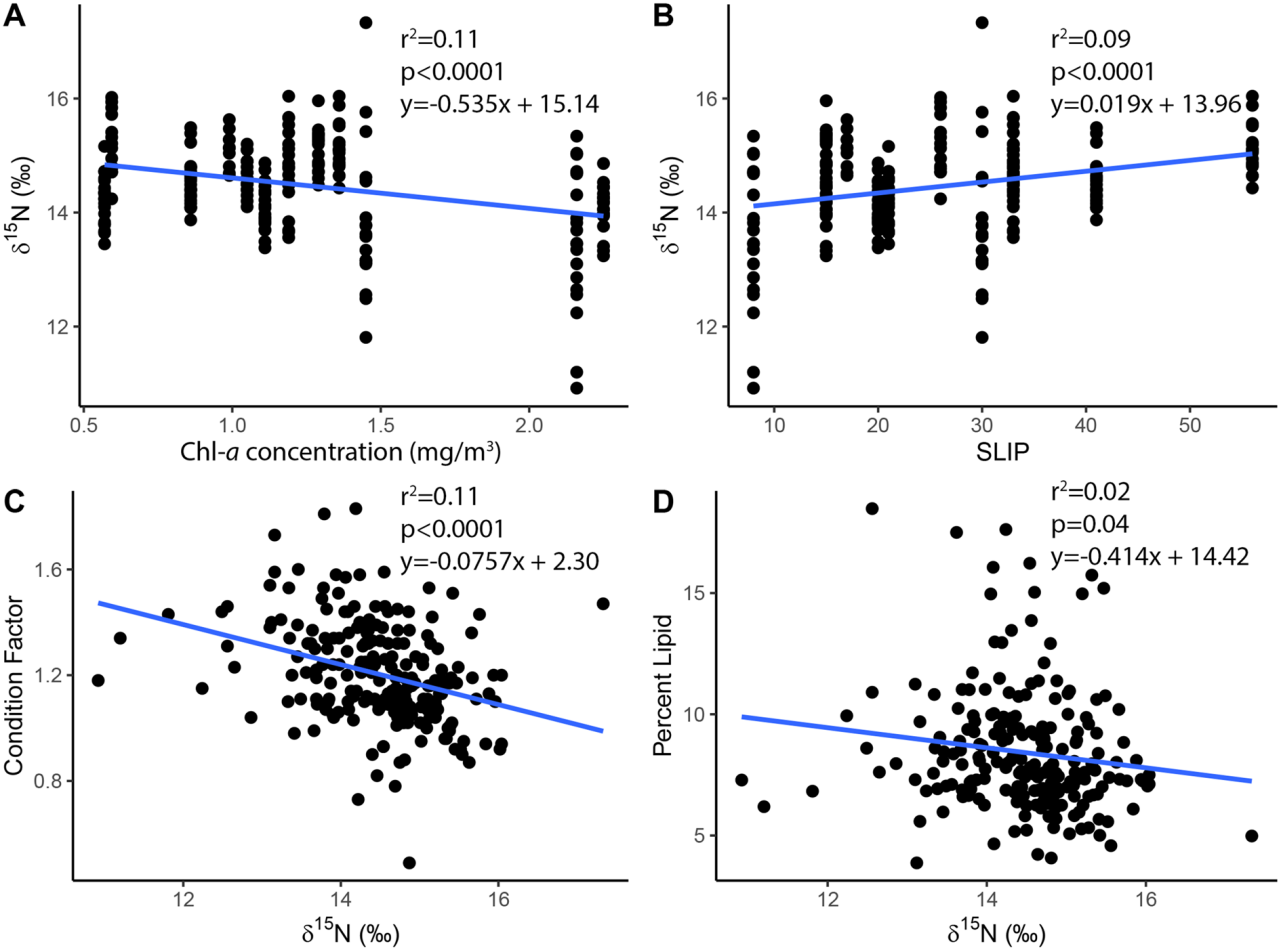
Influence of environmental conditions on Arctic char δ^15^N (**A,B**) and influence of Arctic char δ^15^N on condition factor and percent lipid (**C,D**) in Nain, Labrador, Canada. Lower δ^15^N in Arctic char was associated with higher chlorophyll *a* (Chl-*a*) concentrations (mg/m^3^) (**A**). Higher δ^15^N in Arctic char was associated with a longer Seasonal Loss of Ice Period (SLIP) (**B**). Lower condition factor (**C**) and percent lipid (**D**) in Arctic char was associated with higher δ^15^N.

Environmental variables within Arctic char and ringed seal home ranges are summarized in Tables 3 and 4, respectively. Within the home range of Arctic char there was an increase in seasonal loss of ice period (SLIP) and a decrease in chlorophyll *a* concentrations that were correlated with Arctic char δ^15^N values over the study period (Table 5). We found a negative relationship between Arctic char δ^15^N values and chlorophyll *a* concentration (p < 0.0001, R^2^ = 0.11), whereas we found a positive relationship with Arctic char δ^15^N values and SLIP (p < 0.0001, R^2^ = 0.21) (Fig. 5). Additionally, we found a negative relationship between δ^15^N and condition factor (p < 0.0001, R^2^ = 0.11) and δ^15^N and percent lipid (p = 0.04, R^2^ = 0.02) (Fig. 5).

**Table 3:** Environmental variables, day of opening (DOO, ordinal day of year), day of retreat (DOR, ordinal day of year), seasonal loss of ice period (SLIP, ordinal day of year), maximum chlorophyll *a* concentration (mg/m^3^), and summer sea surface temperature (°C) measured within the home range of Arctic char in Labrador, Canada.

Year	Day of opening (DOO)	Day of retreat (DOR)	Seasonal loss of ice period (SLIP)	Maximum chlorophyll *a* concentration (mg/m^3^)	Summer sea surface temperature (°C)
2006	99	132	33	1.05	6.57
2007	156	177	21	0.57	5.72
2008	148	163	15	2.25	7.08
2009	167	175	8	2.16	5.90
2010	147	177	30	1.45	7.32
2013	156	197	41	0.86	5.83
2014	152	172	20	1.11	7.02
2015	124	157	33	1.19	5.81
2016	164	179	15	1.29	6.10
2017	149	166	17	0.99	6.16
2018	128	184	56	1.36	4.90
2019	148	174	26	0.59	6.10

**Table 4:** Environmental variables, day of opening (DOO, ordinal day of year), day of retreat (DOR, ordinal day of year), seasonal loss of ice period (SLIP, ordinal day of year), maximum chlorophyll *a* concentration (mg/m^3^), and summer sea surface temperature (°C) measured within the home range of ringed seals in Labrador, Canada.

Year	Day of opening (DOO)	Day of retreat (DOR)	Seasonal loss of ice period (SLIP)	Maximum chlorophyll *a* concentration (mg/m^3^)	Summer sea surface temperature (°C)
2002	163	186	23	1.45	3.78
2003	124	164	40	1.37	5.22
2004	90	159	69	1.56	4.67
2005	86	162	76	1.47	4.36
2006	109	158	49	1.23	4.83
2007	138	171	33	1.42	4.19
2008	124	167	43	1.34	5.13
2009	135	179	44	1.66	4.13
2010	140	168	28	1.61	5.47
2011	64	149	85	1.54	4.77
2012	127	168	41	1.44	5.23
2013	120	178	58	1.30	3.79
2014	147	172	25	1.66	4.70
2015	103	172	69	1.74	3.95
2016	142	179	37	1.69	4.10
2017	128	172	44	1.54	4.04
2018	116	179	63	1.81	3.38
2019	132	161	29	1.52	4.41
2020	75	168	93	1.25	5.16

**Table 5:** Environmental variables within the Arctic char home range, with day of retreat (DOR) and seasonal loss of ice period (SLIP) increasing over time and chlorophyll *a* concentrations and summer sea surface temperature decreasing over time.

Independent variable	Response variable	Equation of the line	p-value	R^2^
Year	Day of opening (DOO)	y = 0.472 x – 805.8	0.122	0.01
Year	Day of retreat (DOR)	y = 1.498 x – 2842	< 0.0001	0.16
Year	Seasonal loss of ice period (SLIP)	y = 1.025 x – 2036	< 0.0001	0.11
Year	Maximum chlorophyll *a* concentration (mg/m^3^)	y = − 0.032 x + 65.97	< 0.0001	0.07
Year	Summer sea surface temperature (°C)	y = − 0.062 x + 131.6	< 0.0001	0.16

**Figure 6:**
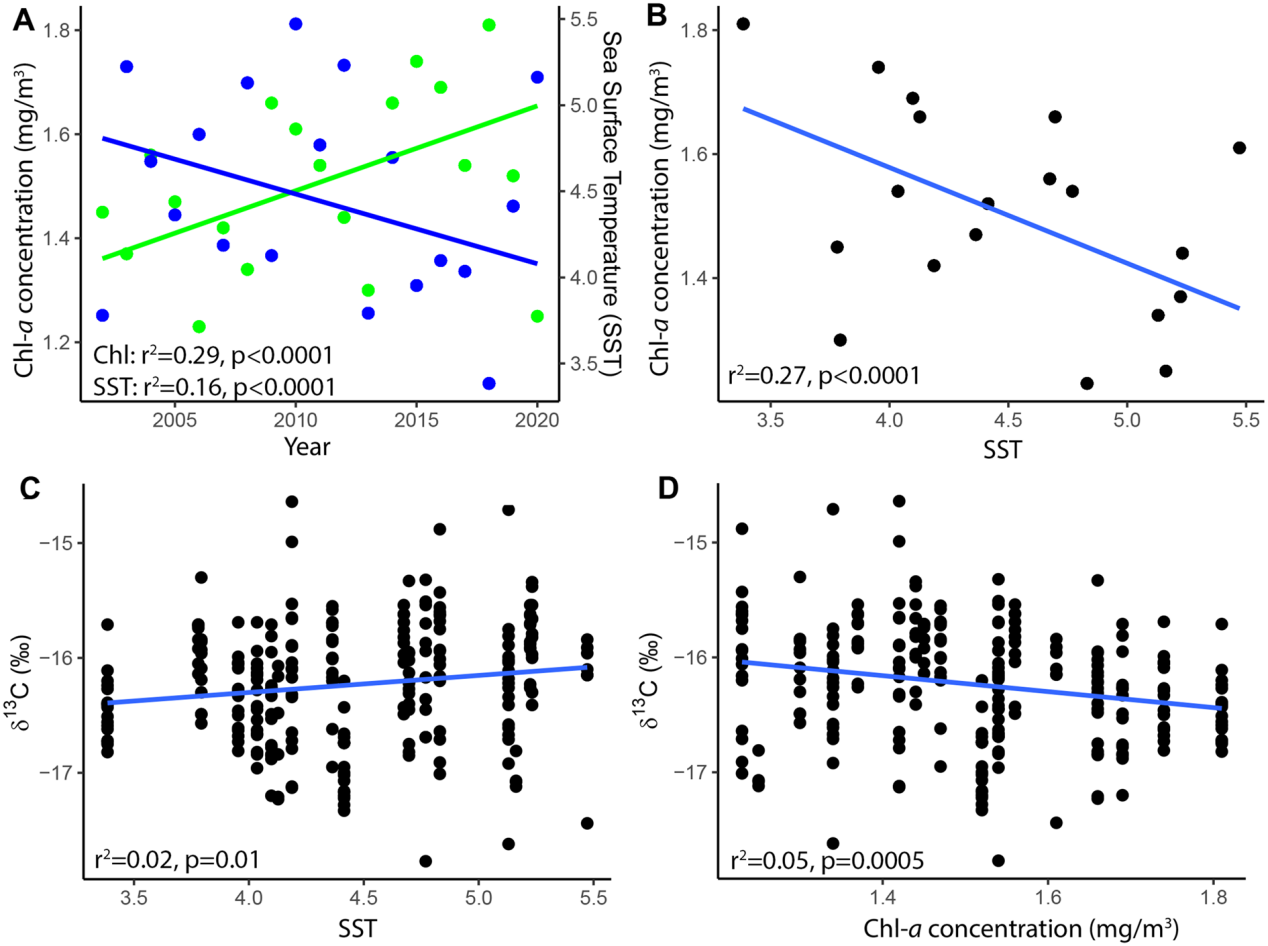
Rising chlorophyll *a* (Chl-*a*) concentrations (green) and decreasing summer sea surface temperatures (SST, blue) measured within northern Labrador ringed seal home ranges from 2002 to 2020 (**A**). A decrease in Chl-*a* concentrations was found in years with greater SST (**B**). Ringed seals fed more pelagically (δ^13^C) with lower SST and higher Chl-*a* concentrations (**C,D**).

Within ringed seal home range we observed an increase in chlorophyll *a* concentrations (p < 0.0001, R^2^ = 0.29) and a decrease in SST (p < 0.0001, R^2^ = 0.16) over the 19 year study period that were correlated with ringed seal δ^13^C values (Table 6, Fig. 6A). Additionally, we found that chlorophyll *a* concentration decreased with increasing SST (p < 0.0001, R^2^ = 0.27, Fig. 6B).

**Table 6:** Environmental variables within the ringed seals home range, with day of retreat (DOR) and maximum chlorophyll *a* concentration (mg/m^3^) increasing over time and summer sea surface temperature decreasing over time.

Independent variable	Response variable	Equation of the line	p-value	R^2^
Year	Day of opening (DOO)	y = 0.467 x – 820.0	0.081	0.01
Year	Day of retreat (DOR)	y = 0.433 x – 702.5	< 0.0001	0.07
Year	Seasonal loss of ice period (SLIP)	y = -0.034 x + 117.5	0.875	0.00
Year	Maximum chlorophyll *a* concentration (mg/m^3^)	y = 0.016321 x – 31.31	< 0.0001	0.29
Year	Summer sea surface temperature (°C)	y = −0.040499 x + 85.89	< 0.0001	0.16

We observed a positive relationship between ringed seal δ^13^C values and SST (p = 0.01, R^2^ = 0.02, Fig. 6C), whereas we observed a negative relationship between δ^13^C values and chlorophyll *a* concentration (p = 0.0005, R^2^ = 0.05, Fig. 6D). We found no relationship between ringed seal isotopic niche area and any of the environmental variables.

## Discussion

Temporal trends of stable isotopes in Arctic char and ringed seals indicate that these two Arctic predators have undergone shifts in diet over the past two decades along the Labrador coast of Canada, which were related to changes in environmental conditions, particularly sea ice, sea surface temperature, and primary productivity. Carbon sources, based on δ^13^C, have become more pelagic/offshore in ringed seals, which likely reflects increasing seasonal open water primary productivity and is consistent with studies on this species from other areas of the Canadian Arctic^9,50^. No change in carbon sources based on δ^13^C in Arctic char were found, suggesting a continued reliance on prey with similar δ^13^C. Marine resources, based on δ^34^S, have increased in Arctic char. This increase likely reflects a combination of dietary choices, with an increase in capelin consumption as their abundance has increased^51^, and greater time spent in the marine environment associated with longer open water periods. These findings are consistent with recent studies on Arctic char in Cumberland Sound^48^. Both species showed increasing δ^15^N over time, suggesting feeding at a higher trophic position that could reflect greater consumption of forage fish, particularly capelin, whose populations have expanded northward. An extreme year for poor sea ice conditions because of variable ice coverage and abnormally high SSTs in 2010 resulted in large variability in stable isotopes in both species and values of isotopic niche volume in Arctic char that was orders of magnitude greater than the yearly mean value reflecting the dietary plasticity of both species. Despite this highly variable feeding behavior in 2010, the condition factor of Arctic char sampled was the highest of all years sampled.

While there were no temporal trends in Arctic char δ^13^C, there was variability between years and a significant shift to more pelagic resources (lower δ^13^C) in 2009 and 2010, when there was an abnormally long period of open water, poor ice conditions and high summer SSTs. The lack of a temporal trend in carbon source for the Arctic char may reflect a strong tie to feeding on pelagic forage fish [e.g., capelin, sand lance (*Ammodytes *spp.)] and zooplankton and little use of benthic resources^10,51^. Further, changes in water column primary productivity associated with ice changes may not have been large enough over the study period to significantly alter temporal trends in Arctic char carbon sources. Within Arctic marine food webs, there has been more pelagic feeding because of greater productivity compared to benthic regions^52^. This increase in primary productivity may help support large amounts of pelagic zooplankton that provide feeding opportunities to Arctic char. The large shift to more pelagic resources in 2010 suggests that continued reduction in sea ice could shift carbon sources for Arctic char, and potentially for other species consuming pelagic prey^9,52^. Such a large change in Arctic char isotopes for a single year does suggest an ability for this species to adapt their diet in the face of large environmental change. While Arctic char can dramatically change their feeding behavior for a given year, this does not necessarily mean that these changes are sustainable for Arctic char over a more extended period of time.

Labrador coast ringed seals shifted to a more pelagic diet over the past 18 years, based on decreasing trends in δ^13^C between 2002 and 2020, consistent with populations in other areas of the Arctic, including Hudson Bay and the Beaufort Sea^9,50^. Chlorophyll *a* concentration and SST were environmental drivers of δ^13^C in ringed seals. Both these environmental variables had a temporal trend over the study period. Increased chlorophyll *a* concentrations support a stronger phytoplankton carbon pathway^53^, explaining the shift in ringed seal feeding to more pelagic prey. Lower summer SSTs that occurred over the study period, possibly influenced by influxes of cold meltwater from the Arctic^54^, allow for greater phytoplankton abundance and grazing by *Calanus* sp.^55^ supporting more pelagic feeding by ringed seals. This phytoplankton carbon pathway includes forage fish, such as capelin and sand lance^11^, that are high in nutritional content and are proving to be an important component of ringed seal diets^33,56^.

Increasing δ^15^N in both Arctic char and ringed seals indicates that these species are feeding at higher trophic positions over the study period. This likely reflects an increase in forage fish consumption, namely capelin, that have become more abundant in the Arctic as it has warmed^48,57^. However, others found that δ^15^N did not increase in Arctic char in Cumberland Sound, despite capelin becoming a significant component of their diet^48^. Capelin abundance is largely driven by bottom-up regulation and is heavily influenced by sea ice conditions and primary productivity^58^. The capelin abundance along the Labrador coast has been increasing since the crash in the early 1990’s, and long-term commercial catches indicate that char habitat use and diet near Nain is varying annually in relation to capelin availability^51^. Arctic char have been shifting to a more piscivorous diet along the Labrador coast, driving this increase in trophic position, as forage fish occupy a higher trophic position than marine invertebrates^10^. Arctic char trophic position in this study was also higher in years with low chlorophyll *a* concentrations and when it takes longer for sea ice to completely break up. No temporal trends in the trophic position of ringed seals were observed in Greenland or Hudson Bay, although, consistent with our findings these studies found that higher ringed seal trophic position was associated with greater occurrence of capelin in diet^21,59^. Additional research is needed to better understand the relationship between δ^15^N and trophic position of Arctic char and ringed seals in Labrador, and piscivorous fish and marine mammals in general in a changing Arctic food web.

The use of marine resources has increased over the time of this study in Arctic char, reflected in increasing δ^34^S, indicating longer feeding periods and greater resource use in marine areas. Arctic char utilized more marine resources when the sea ice break up period (SLIP) took longer. These variable sea ice break up conditions may influence char to either enter the marine environment earlier and/or spend more time offshore. There was also a strong correlation between Arctic char δ^15^N and δ^34^S, which suggests that this shift to more marine resources is also associated with feeding at a higher trophic position. There is evidence that as Arctic char in northern Labrador feed more offshore, they consume more capelin^51^, which may result in higher δ^15^N and δ^34^S in their tissues. There has been limited use of δ^34^S in studies of Arctic char, but as demonstrated here, it can be useful in characterizing complex feeding behaviors of this anadromous species.

While, Arctic char isotopic niche size did not have a temporal trend, it was significantly larger in 2009 and 2010 compared to the other years. This large isotopic niche volume reflects the ability of individual Arctic char to adapt their diet within a year, reflecting their dietary plasticity as well as capability to adjust to abnormal environmental conditions. Similar to Arctic char, ringed seals had a larger isotopic niche area in 2010, where it was the second largest amongst all years. These findings suggest that there may have been ecosystem wide changes in the food web during this poor ice year with higher SST compared to other years. Despite there being changes in ringed seal isotopic niche area for this particular year, there were no temporal trends over our entire study period, which is consistent with the absence of temporal trends of isotopic niche area for ringed seals in Hudson Bay^9^.

The condition and lipid content of Arctic char decreased over the 13-year sampling period. These trends were commensurate with environmental conditions and the feeding behavior of Arctic char. Arctic char δ^15^N decreased with increasing chlorophyll *a* concentration and increased with SLIP. There was a negative relationship between SLIP and chlorophyll *a* concentration, possibly explained by intermittent ice coverage and limited light availability resulting in weak phytoplankton blooms. With a weak phytoplankton bloom, there is less food for pelagic invertebrates which may lead to Arctic char feeding on higher trophic level species, as well as an overall lower amount of prey biomass available with feeding higher in the food web. The shift in Arctic char feeding on higher trophic level species was connected to declines in condition factor and lipid content (Fig. 5). This reduction in char condition may also reflect reduced nearshore abundance or quality of preferred prey, leading to an increased energy expenditure to access offshore food resources. As there is a positive relationship between Arctic char δ^15^N and δ^34^S, there is a possibility that Arctic char have to travel further for their prey, exerting more energy traveling away from their freshwater habitat and into the marine environment.

Dietary shifts in Labrador Arctic char and ringed seals may be expected as climate change introduces new species into more northern regions with corresponding changes in competitive and predatory interactions altering food web structure^60^. Capelin moving in from the south have been shown to alter the predative interactions of beluga whales on Greenland halibut, with beluga whales shifting their feeding to capelin during the summer months^13^. An increase in trophic position for both species and a shift to more pelagic feeding by ringed seals, Closer to the carbon signatures that are found in Arctic char, could result in these two species competing more for food resources in the future. As both Arctic char and ringed seals shift to feeding at a higher trophic position, this may lead to increased concentrations of biomagnifying contaminants, such as mercury (Hg) and polychlorinated biphenyls (PCBs) over time^61^.

Climate warming and associated changes in ice and marine productivity within the Arctic may result in a shift in marine food webs and the invasion of subarctic species such as Atlantic salmon (*Salmo salar*)^43,62^. Our study provides evidence that both Arctic char and ringed seals have altered their feeding ecology over a 13- and 18-year period, respectively, in response to interannual variability in environmental conditions associated with climate warming. The parallel (i.e., δ^15^N trends) and contrasting (i.e., δ^13^C trends) changes observed across both species exemplify how species vary in their response to environmental change, which may have significant implications for shifts in competition/predation in Arctic marine food webs. These findings emphasize the importance of continued monitoring of stable isotope profiles in these valued and circumpolar species, as well as the need to improve our understanding of what is driving these shifts, particularly potential changes at lower trophic levels.

## Methods

### Sample collection

All samples were collected from subsistence-harvested anadromous Arctic char and ringed seals. Arctic char (*n* = 214, Table 1) were collected from around Nain from August–September (2006–2010 and 2013–2019, 12 years of data spanning 13 years) before they migrate back to freshwater to overwinter. Ringed seals (*n* = 53, Table 2) were harvested from three marine inlets in northern Labrador (Nachvak, Okak, and Saglek; 2008–2011 and 2019–2020) from September–October (Fig. 1). The 2008–2011 and 2019–2020 ringed seal claws provided a record from 2002–2011 and 2009–2020, respectively, which spanned an 18-year period.

Arctic char fork length (mm), round weight (g), and sex were recorded, muscle was taken near the dorsal fin and frozen until analysis. Otoliths were extracted for aging and analyzed by AAE Tech Services (La Salle, Manitoba, Canada). Following harvesting, ringed seal total length and girth were recorded, and the lower jaw and left fore flipper was extracted. Teeth were aged at Matson's Laboratory, USA, by longitudinally thin sectioning a lower canine tooth and counting annual growth layers in the cementum using a compound microscope and transmitted light. Flippers were frozen at −20 °C before having the digit I claw extracted for stable isotope analysis.

### Stable isotope analysis

Dorsal muscle tissue from Arctic char was freeze dried at −48 °C and 133 × 10^3^ mbar for 48 h. Dried muscle was crushed into a fine powder using surgical scissors and 400–600 µg of muscle tissue was weighed into tin capsules. Only adult ringed seals (≥ 6 years old) were used as they are known to undergo an ontogenetic shift in diet when they reach sexual maturity^18,63^. Claws from the first digit of the left fore flipper were removed using a scalpel for stable isotope analysis. The claw was detached from the bone by placing it in a water bath at 60 °C, cleaned using ethanol, and stored in deionized water^42^. Ringed seal claws can include up to 10 years of growth bands. Individual light and dark claw bands were cut using a surgical scalpel and 400–1000 µg of claw bands were weighed into tin capsules. Tin capsules with Arctic char muscle and ringed seal claw bands were analyzed for δ^13^C and δ^15^N by Delta V Thermoscientific Continuous Flow Mass Spectrometer (Thermo Scientific, Bremen, Germany) coupled to a 4010 Elemental Combustion System (Costech Instruments, Valencia, CA, USA). Muscle and nail band tissue was also weighed (5500–6000 µg) for δ^34^S into capsules and analyzed on a Delta V Plus Thermoscientific Continuous Flow Mass Spectrometer (Thermo Scientific, Bremen, Germany) coupled to a 4010 Elemental Combustion System. Isotopic ratios were reported as:

$$\delta {\text{X }} = \, \left[ {\left( {{\text{Rsample}}/{\text{Rstandard}}} \right) \, - { 1}} \right]$$

where, X is either ^13^C, ^15^N or ^34^S, R is the ratio ^13^C/^12^C, ^15^N/^14^N or ^34^S/^32^S, and the standards used were C from Vienna Peedee Belemnite (VPDB), N from atmospheric, or S from the Canyon Diablo troilite (CDT). The analytical precision [standard deviation (SD)] for NIST standard 1577c (bovine liver), an internal laboratory standard (tilapia muscle), USGS 40 and Urea (n = 50 for all) for δ^13^C and δ^15^N were < 0.20‰. The analytical precision for δ^34^S values from NIST 1577c, an internal laboratory standard, USGS 42, NIST 8555 and NIST 8529 (n = 118 for all) was < 0.25‰. The accuracy based on the certified USGS 40 sample (n = 50) showed a difference of 0.13 and -0.02‰ of the mean calculated values for δ^13^C and δ^15^N, respectively. NIST standards 8573, 8547, and 8574 for δ^15^N and 8542, 8573, 8574 for δ^13^C (n = 10 for all) were used to check the accuracy of the stable isotope analyses. The mean difference from the certified values were –0.09, 0.14, −0.06‰ for δ^15^N and 0.09, 0.01, and −0.08‰ for δ^13^C respectively. For δ^34^S, the accuracy using USGS 42 (n = 118) was within 0.12‰ of the mean calculated value.

### Arctic char lipid extraction analysis

Arctic char lipid percentage was determined on dried muscle tissue by performing a series of lipid extractions using a 2:1 mixture of chloroform: methanol using methods outlined in Bligh and Dyer^64^.

### Environmental variables

Environmental data were extracted in a Geotiff format within Arctic char and ringed seals home ranges as described in^65^ and^66^, respectively (Fig. 1) using QGIS (version 3.30.3, QGIS Development Team)^67^. Environmental data was extracted for 2006–2010 and 2013–2019 within the Arctic char home range and 2002–2020 within the ringed seal home ranges. The area used for Arctic char was based on migratory distribution, which is within 100 km of the catch location and proximity of their resident rivers ^65^, whereas for ringed seals the area was 196,886 km^2^ and was based on the minimal convex polygons of 95% of their space use ^66^.

Daily sea ice concentration (SIC), collected remotely from satellites, were acquired from the Sea Ice Index database at the National Snow and Ice Data Center website (https://nsidc.org/data/explore-data) at a 25 km × 25 km resolution and were used to determine the date of sea ice break up. Arctic sea ice is highly variable between years and dates can be selected to summarize sea ice retreat by using sea ice concentration^68^. We utilized a number of variables to characterize spring sea ice break up across species home ranges, including: day of opening (DOO), the last day SIC drops below 80% within the 25 km × 25 km pixel; day of retreat (DOR), the last day SIC drops below 15%; and, seasonal loss of ice period (SLIP), which is the number of days between DOO and DOR. Break up date was determined when 50% of the sampling points within the home range for ringed seals and Arctic char dropped below a variable’s defined SIC percentage.

Weekly chlorophyll *a* concentration (mg/m^3^) collected from AQUA/MODIS satellites from 2002 to 2020 was retrieved from the Nasa Earth Observations website at a 0.25° resolution. Some cells did not have chlorophyll *a* concentrations for a given week because of interference from cloud cover or sea ice. Since it was not possible to differentiate between cloud cover and sea ice for cells with no data, extrapolation was not attempted. Instead, mean chlorophyll *a* concentration was calculated based on the available data for that given week and was accepted for analysis if greater than 50% of the sampling points had data and was a week that followed sea ice break up. Weeks that had less than 50% sampling points with data were excluded as to not incur bias based on a limited number of sampling points with data. The week with the maximum chlorophyll *a* concentration was chosen to represent when the peak phytoplankton bloom occurred as this triggers periods of intense summer feeding.

Monthly SST (C) collected from AQUA/MODIS satellites from 2002 to 2020 was retrieved from the Nasa Earth Observations website at a 0.25° resolution to analyze summer SST following sea ice break-up. The data format was similar to the chlorophyll *a* data but at a monthly temporal resolution and extracted in the same manner as sea ice using QGIS. Mean summer SST was calculated using July, August, and September months as they are months with open water. The influence that summer SST has on feeding ecology of Arctic char and ringed seals was assessed during this period as this is when significant feeding takes place.

### Statistical analyses

Statistical analyses were performed using RStudio (version 4.1.1, R Core Team 2021)^69^. Sex, age, fork length, round weight, and Fulton’s condition factor (based on fork length and weight using (K = 100 × weight/length^3^)^47^ of Arctic char were analyzed to determine which variables needed to be accounted for prior to temporal and environmental trend analysis of stable isotopes. The influence of sex on length, weight, condition factor, and stable isotopes (δ^13^C, δ^15^N, or δ^34^S) was assessed using two-sample t-tests. The influence of age on stable isotopes (δ^13^C, δ^15^N, or δ^34^S) was assessed by a one-way ANOVA, followed by Tukey tests to identify ages that differed. The influence of year on fork length and round weight of Arctic char was assessed by a one-way ANOVA, followed by Tukey tests to identify years that differed. As fish size varied by year, this highlighted the importance to assess the possible trends with size and δ^13^C, δ^15^N, and δ^34^S for each year using linear regression.

Location, sex, and age of ringed seals were analyzed to determine what factors needed to be accounted for prior to temporal and environmental trend analysis of δ^13^C and δ^15^N. The influence of location on stable isotopes (δ^13^C or δ^15^N) was assessed using one-way ANOVA. The influence of sex on stable isotopes (δ^13^C or δ^15^N) was assessed using two-sample t-tests. The influence of ringed seal age, as determined by the annuli along the claw, on stable isotopes (δ^13^C or δ^15^N) was assessed using linear regression. An age correction for ringed seal δ^15^N was required to standardize across ages as this stable isotope increased linearly with age, where:

$${\text{Age adjusted }}\updelta^{{{15}}} {\text{N}}\, = \,\updelta^{{{15}}} {\text{N}}{-}\left( {{\text{m }}*{\text{ Age}}} \right),$$

where m is the slope of the relationship between age and δ^15^N.

No temporal trends (2002–2011) and little to no variability between years was observed for δ^34^S in the claws of ringed seals collected from 2008 to 2011, which is consistent with their predominant marine distribution and marine foraging behaviour^18^, therefore, claws collected in 2019–2020 were not analyzed for δ^34^S. Ringed seal condition was only determined for 2008 and 2019 by ((maximum girth (cm)/total length (cm)) × 100)^18^, as seals were not collected in most other years. Ringed seal condition between 2008 and 2019 was assessed using two-sample t-tests as these were the years when the majority of ringed seals were sampled (n = 13; n = 14). The influence of age on condition factor was also assessed for both years using linear regression and no trends (p > 0.05) were found.

### Isotopic niche size

The R package NicheROVER^49^ is capable of calculating an isotopic niche region beyond two dimensions and was used to calculate the isotopic niche volume of Arctic char using δ^13^C, δ^15^N and δ^34^S. The niche region is defined as the 95% probability region in multivariate space^49^. The influence of year on isotopic niche volume of Arctic char was assessed by a one-way ANOVA. Ringed seal isotopic niche area was calculated using δ^13^C and δ^15^N with Stable Isotope Bayesian Ellipses in R (SIBER)^38^ to calculate the standard ellipse area at a 95% probability using two stable isotopes, as δ^34^S did not vary between years for ringed seals. The influence of year on isotopic niche volume of ringed seals was assessed by a one-way ANOVA. As there were three stable isotopes used for Arctic char and two for ringed seals, isotopic niche size will be differentiated by volume and area for the respective species.

### Temporal trend analysis

Calanus copepods from 2010 and 2020 were analyzed for δ^15^N to test for shifts in baseline isotopic values over time and assessed using two sample t-test. Relationships were assessed between δ^13^C, δ^15^N, δ^34^S, condition factor, percent lipid, and isotopic niche volume with year for Arctic char using linear regression. Relationships between δ^13^C, age corrected δ^15^N, and isotopic niche area with year for ringed seals was assessed using linear regression. Ringed seal condition was only determined for 2008 and 2019 by ((maximum girth (cm)/total length (cm)) × 100)^18^, as seals were not collected in most other years. Ringed seal condition between 2008 and 2019 was assessed using two-sample t-tests as these were the years when the majority of ringed seals were sampled (n = 13; n = 14).

### Relationships between environmental parameters and Arctic char and ringed seal feeding ecology

Linear regression was used to examine relationships between environmental parameters (DOO, DOR, SLIP, SST, and chlorophyll *a* concentration) and Arctic char stable isotopes (δ^13^C, δ^15^N, δ^34^S), isotopic niche volume, condition factor, and percent lipid. Similarly, linear regression was used to examine the relationship between environmental parameters (DOO, DOR, SLIP, SST, and chlorophyll *a* concentration) and ringed seal stable isotopes (δ^13^C and age-adjusted δ^15^N) and isotopic niche area. Linear regression plots were developed to display significant relationships between environmental explanatory variables and both Arctic char and ringed seal biological response variables.

## Data Availability

The datasets used and/or analysed during the current study are available from the corresponding author on reasonable request.
